# The effect of HPV DNA and p16 status on the prognosis of patients with hypopharyngeal carcinoma: a meta-analysis

**DOI:** 10.1186/s12885-022-09769-w

**Published:** 2022-06-15

**Authors:** Jinfeng Shi, Ling Wang, Nan Yao, Le Sun, Wenyu Hu, Xiaotong Li, Yixue Yang, Yusheng Wang, Wei Zhu, Bo Li

**Affiliations:** 1 Department of Otolaryngology Head and Neck Surgery, The First Hospital of Jilin University, Xinmin Street No. 71, JiLin 130021 Changchun, P.R. China; 2 Department of Epidemiology and Biostatistics, School of Public Health, Jilin University, Xinmin Street 1163, 130021 Changchun, P. R. China

**Keywords:** Hypopharyngeal Carcinoma (HPC), Human papilloma virus (HPV), p16, Prognostic, Meta-analysis

## Abstract

**Objective:**

To evaluate whether the presence of human papillomavirus (HPV) DNA and p16 might be associated with better prognosis in patients with hypopharyngeal carcinoma (HPC), especially on overall survival (OS) and disease-free survival (DFS).

**Method:**

PubMed, the Cochrane Library, the Web of Science and EMBASE were searched from inception to April 2021 to search for HPV DNA- and p16-related prognostic articles on HPC. Meta-analysis was performed on the selected articles according to the inclusion and exclusion criteria. Publication bias was assessed for the included studies with Egger’s test. All studies were analyzed by using Stata 16.0 statistical software.

**Results:**

A total of 18 studies were included, including 12 HPV DNA studies and 11 p16 studies. Meta-analysis showed that HPV DNA positivity was a strong prognostic factor for improved OS in patients with HPC, with a pooled hazard ratio (HR) of 0.61 (95% CI, 0.54–0.69), but there was no statistically significant difference in DFS (HR, 0.60; 95% CI, 0.31–1.16). Patients with p16-positive tumors had better OS (HR, 0.66; 95% CI, 0.49–0.89) and DFS (HR, 0.59; 95% CI, 0.44–0.78) than patients with p16-negative tumors.

**Conclusions:**

This study suggests that the presence of HPV DNA leads to better OS in patients with HPC, and the presence of p16 also corresponds to better OS and DFS. Our results provide up-to-date evidence to clinicians and researchers. Larger studies adjusting for prognostic factors are needed in subsequent studies.

**Supplementary Information:**

The online version contains supplementary material available at 10.1186/s12885-022-09769-w.

## Introduction

Hypopharyngeal carcinoma (HPC) is a relatively rare type of tumor and comprises less than 5% of all head and neck squamous cell carcinomas (HNSCCs) [1]. However, due to its aggressiveness and hidden location, HPC is often diagnosed at an advanced stage. The operative and postoperative treatment of HPC are difficult, resulting in a relatively low overall survival rate, so it is considered one of the head and neck tumors with poor prognosis [2].

Human papillomavirus (HPV) is a type of DNA virus that can infect human mucosa and skin, and it is related to the occurrence of many human diseases. Since HPV DNA was first detected in human head tissue [3], the relationship between HPV infection and HNSCC has received more attention. In addition to HPV, p16 as an immunohistochemical (IHC) marker, has potential value in the prediction of prognosis in some cancers. Based on the integration of viral DNA leads to interruption of E2, a silencer of viral DNA translation, thus leading to enhanced E6 and E7 oncogene production. The latter (E7) again interferes with cellcycle regulation leading to overexpression of an inhibitor of cdk, namely p16. Therefore, overexpression of p16 measured by p16 immunohistochemistry, has been considered as a surrogate marker of oncogenic HPV infection in many studies [4–6]. However, studies have found that there was a certain degree of mismatch between P16 immunohistochemistry and HPV DNA detection methods [7]. Therefore, in order to gain insight into the impact of HPV and p16 on cancer, many studies have chosen to analyze HPV and P16 separately [8, 9].

Interestingly, HPV is not only a risk factor for some diseases but may also be a good predictor of some cancers. Multiple studies have reported that HPV is a positive prognostic indicator in oropharyngeal squamous cell carcinoma (OPSCC), and the risk of death in HPV-positive OPSCC patients was reduced by approximately half compared to patients with HPV-negative tumors [10–12]. In addition, p16 has also been suggested as a prognostic factor in head and neck squamous cell carcinoma. In OPSCC, patients with p16 over expressing tumors have shown better diseases-free survival (DFS) compared to tumors which lack p16 expression [13, 14]. Meanwhile, some studies have found that p16 expression was identified as an independent prognostic factor associated with OS and DSS in laryngeal squamous cell carcinoma [15, 16].

However, the effect of either HPV or p16 status on prognosis in HPC has not been clearly concluded. To date, many studies have examined the relationship between HPV infection and the prognosis of HPC, with some suggesting that HPV-positive HPC patients have a better prognosis [17, 18]. Some research, however, has shown that there is no obvious correlation between HPV infection and the prognosis of HPC [19, 20], and some studies even drew opposite conclusions [21]. Similarly, the relationship between p16 and HPC prognosis is also unclear.

To our knowledge, it has not been determined whether HPV and p16 status have prognostic significance in HPC as it does in oropharyngeal cancer. This may be due to the low prevalence of HPV- and p16-positive status in hypopharyngeal cancer patients [22, 23], and the results of some studies with small sample sizes may cause confusion with unreliable conclusions. Therefore, the objective of our study was to perform a meta-analysis to examine whether there is a survival advantage of HPV DNA positivity or p16 positivity in HPC patients.

## Materials and methods

### Search strategy and selection criteria

 This meta-analysis was conducted according to the Preferred Reporting Items for Systematic Reviews and Meta-Analyses (PRISMA) guidelines. We performed a systematic literature search in the PubMed, the Cochrane Library, the Web of Science and EMBASE databases. The search was restricted to publications in English. We searched all of the literature up to April 7, 2021 for the combined medical subject headings (MeSH) “Alphapapillomavirus” and “Hypopharyngeal Neoplasms”. The search terms included MeSH terms and their entry terms.

Two researchers independently selected the articles for full review based on the following inclusion criteria: the study population included patients with hypopharyngeal cancer; the article assessed HPV or p16 status in a population with hypopharyngeal cancer; the total number of patients was more than 20; the study measured at least one primary outcome: overall survival (OS), which is generally considered the best efficacy endpoint for oncology clinical trials; or disease-free survival (DFS), which is most commonly used in adjuvant therapy after radical surgery or radiotherapy, is also widely used in prognosis of hypopharyngeal cancer.

The researchers performed a detailed review of the full text of the selected articles. Studies were excluded based on the following criteria: studies that were case reports, reviews, or meta-analyses; studies that included other types of head and neck tumors; HPV was not the prognostic factor; the patients had distant metastatic disease; the exposed group cases were less than 5; studies with insufficient survival data for the calculation of the hazard ratio (HR); studies with low quality assessments; and studies without full-text.

### Data abstraction

Two researchers independently assessed the included articles and conducted the data extraction. If there was a disagreement, help was sought from a third person, Professor Bo Li, who is an expert in evidence-based medicine. Data extraction from each article included (1) first authors, region, year of publication, and year of sample collection; (2) the number of HPV DNA-positive and HPV DNA-negative patients and p16-positive and p16-negative patients; (3) the stage of the cancer in the subjects; (4) the HPV assessment method; p16 testing technique; and (5) the survival outcome (OS or DFS), and hazard ratio (HR) with 95% CIs.

For studies without HRs or CIs, we preferentially extracted their Kaplan–Meier curves using Engauge Digitizer 11.1 software to extract the survival data from their survival curves. Then, we used the method reported by Jayne F. Tierney [24] to estimate HRs and CIs. His article also provided a variety of methods to calculate the HRs for studies without survival curves. Since the HR obtained by some studies was the reverse HR defined by our study, this study also adopted the method used in Tierney’s paper to recalculate the HRs.

### Quality assessment

The Newcastle–Ottawa Scale (NOS) was used to estimate the quality of the included articles. The following information was evaluated in each study: the study population, comparability, and assessment of outcomes. Articles scoring at least 6 out of 9 points were considered high-quality studies. Low scores (score < 6) were excluded from the study.

### Statistical analysis

Meta-analysis was performed using the statistical software Stata version 16.0. We used HRs and their 95% CIs to measure the survival outcomes in the HPV DNA- or p16-positive group compared with the HPV DNA- or p16-negative group. Statistical heterogeneity across studies was evaluated by Cochrane’s Q statistic and I^2^ statistics. If the Q test two-sided P value was < 0.05 or the I^2^ was > 50%, which suggested heterogeneity, we used a random-effect model to calculate the pooled HR. If there was no significant heterogeneity, we applied a fixed-effect model. For the pooled HRs, an HR > 1 suggested that HPV was a risk factor for poor survival, an HR < 1 indicated that HPV infection was a prognostic factor that improved survival, and HR = 1 suggested no significant difference in survival. Since the survival outcomes of the collected studies existed in different years, subgroup analysis of survival indicators in different years will be conducted in this study. A funnel plot and Egger’s test were used to assess publication bias, and we used the trim-and-fill method (sensitivity analysis) to correct outcomes and evaluate the impact of bias on the outcomes. Significance level was set at α = 0.05, all tests were two-sided.

## Results

### Search results

**Fig. 1:**
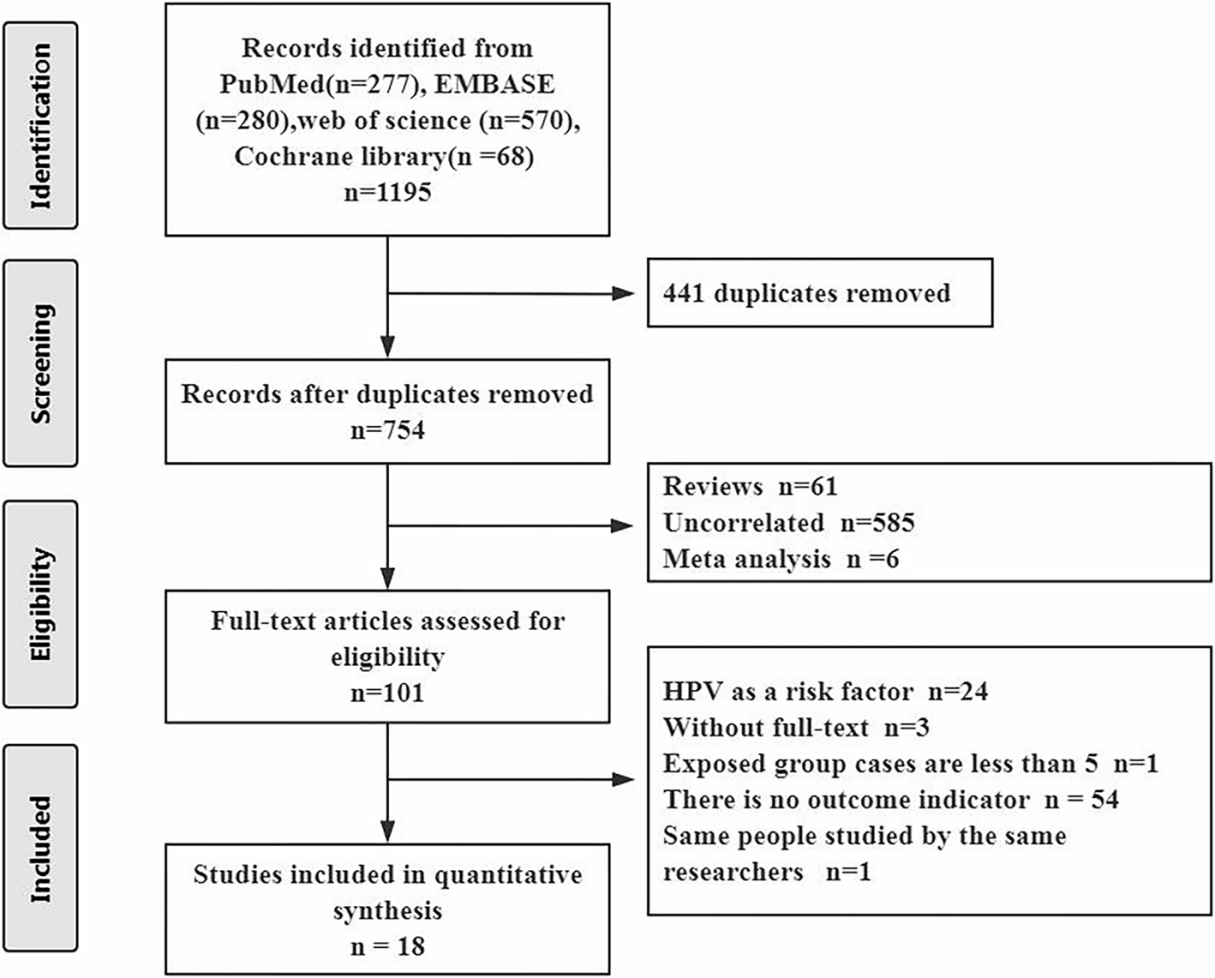
Flow diagram of the literature retrieval and selection for this study

We searched a total of 1195 relevant articles from four databases. After duplicates were excluded, we reviewed the titles and abstracts of 754 articles. According to the inclusion and exclusion criteria, 653 articles were excluded. Finally, by carefully browsing the full text of the remaining 101 articles, we included 18 articles in total [17–21, 25–37]. Among them, 12 articles were related to HPV DNA status, and 11 articles were related to p16 status (Fig. 1).

The characteristics of the 12 included articles on HPV DNA status [17–21, 25–31] and the 11 included articles on p16 status [20, 21, 25, 28, 29, 32–37] are summarized in Tables 1 and 2. All of the studies were published between 2011 and 2020. HPV DNA was detected by polymerase chain reaction (PCR) and in situ hybridization (ISH), whereas the detection methods of four studies were unknown. The HPV genotypes detected in the included studies were shown in Table S1 of the Supplementary materials. All p16 status were detected by immunohistochemistry (IHC) and the definition criteria for p16 positivity in each included study were shown in Table S2 of the Supplementary materials. All studies reported at least one survival outcome in OS and DFS. A total of 6098 patients with HPC were tested for HPV DNA status, and 805 patients with HPC were tested for p16 expression in this meta-analysis. Based on the quality assessment of NOS, all included studies scored higher than six, and no studies were excluded.

**Table 1:** Characteristics of the Included Studies (HPV DNA)

Study	Region	No. of patients	Disease stage, No		HPV diagnostic method	HPV prevalence, %		Outcome	Quality score
			**I-II**	**III-IV**		**Positive**	**Negative**		
Hong et al., 2018^27^	the U.S.	1931	NS	NS	NS	16.9	83.1	5-OS	8
Joo et al., 2013 [30]	Korea	64	NS	NS	ISH	10.9	89.1	5-OS,5-DFS	7
Ernoux et al., 2011 [21]	France	61	0	61	PCR	82.0	18.0	5-DFS	9
Lassen et al., 2017 [28]	Denmark	35	0	35	PCR	14.3	85.7	5-OS	7
Yang et al., 2016 [20]	China	46	9	37	PCR	26.1	73.9	> 5-OS	8
Burr et al., 2018 [19]	the U.S.	63	NS	NS	NS	15.9	84.1	3-OS	7
Dalianis et al.,2015 [29]	Sweden	142	NS	NS	PCR	4.9	95.1	3-OS	7
Marshall et al., 2020 [17]	the U.S.	640	85	555	PCR,ISH	26.1	73.9	3-OS	9
Abdel et al., 2020 [31]	the U.S.	1157	491	666	NS	23.9	76.1	> 5-OS	7
Tian et al., 2019 [26]	the U.S.	1805	165	1640	NS	10.6	49.5	5-OS	8
Joo et al., 2014 [18]	Korea	45	NS	NS	ISH	11.1	88.9	5-OS,5-DFS	7
Wendt et al., 2014 [33]	Sweden	109	21	88	PCR	6.4	93.6	5-OS	7

*Abbreviations: HPV *human papillomavirus, *ISH *in situ hybridization, *NS *not specified, *OS *overall survival, *DFS *disease-free survival, *PCR *polymerase chain reaction.

**Table 2:** Characteristics of the Included Studies (p16)

Study	Region	No. of patients	Disease stage, No		p16 diagnostic method	p16 prevalence, %		Outcome	Quality score
			**I-II**	**III-IV**		**Positive**	**Negative**		
Ernoux et al., 2011 [21]	France	75	0	75	IHC	9.3	90.7	5-DFS	9
Lassen et al., 2017 [28]	Denmark	35	0	35	IHC	14.3	85.7	5-OS	7
Yang et al., 2016 [20]	China	46	9	37	IHC	26.1	73.9	> 5-OS,>5-DFS	8
Dalianis et al., 2015 [29]	Sweden	142	NS	NS	IHC	15.5	84.5	3-OS	7
Lassen et al., 2014 [35]	Denmark	158	0	158	NS	13.3	86.7	5-OS	7
Ang et al., 2015 [37]	Singapore	75	5	70	IHC	6.7	93.3	> 5-OS	7
Lee et al., 2018 [34]	Korea	45	0	45	IHC	24.4	75.6	> 5-OS	7
Wilson et al., 2012 [32]	the U.S.	27	7	20	IHC	33.3	66.7	> 5-OS,>5-DFS	7
Wilson et al., 2014 [24]	the U.S.	32	NS	NS	IHC	34.4	65.6	3-OS,3-DFS	7
Wendt et al., 2014 [33]	Sweden	109	46	63	IHC	16.5	83.5	5-OS,5-DFS	7
Chung et al., 2014 [36]	the U.S.	61	0	61	IHC	16.4	83.6	5-OS	7

*Abbreviations: HPV *human papillomavirus, *IHC *immunohistochemistry, *NS *not specified, *OS *overall survival, *DFS* disease-free survival

### Survival according to HPV DNA status

#### Overall survival

**Fig. 2:**
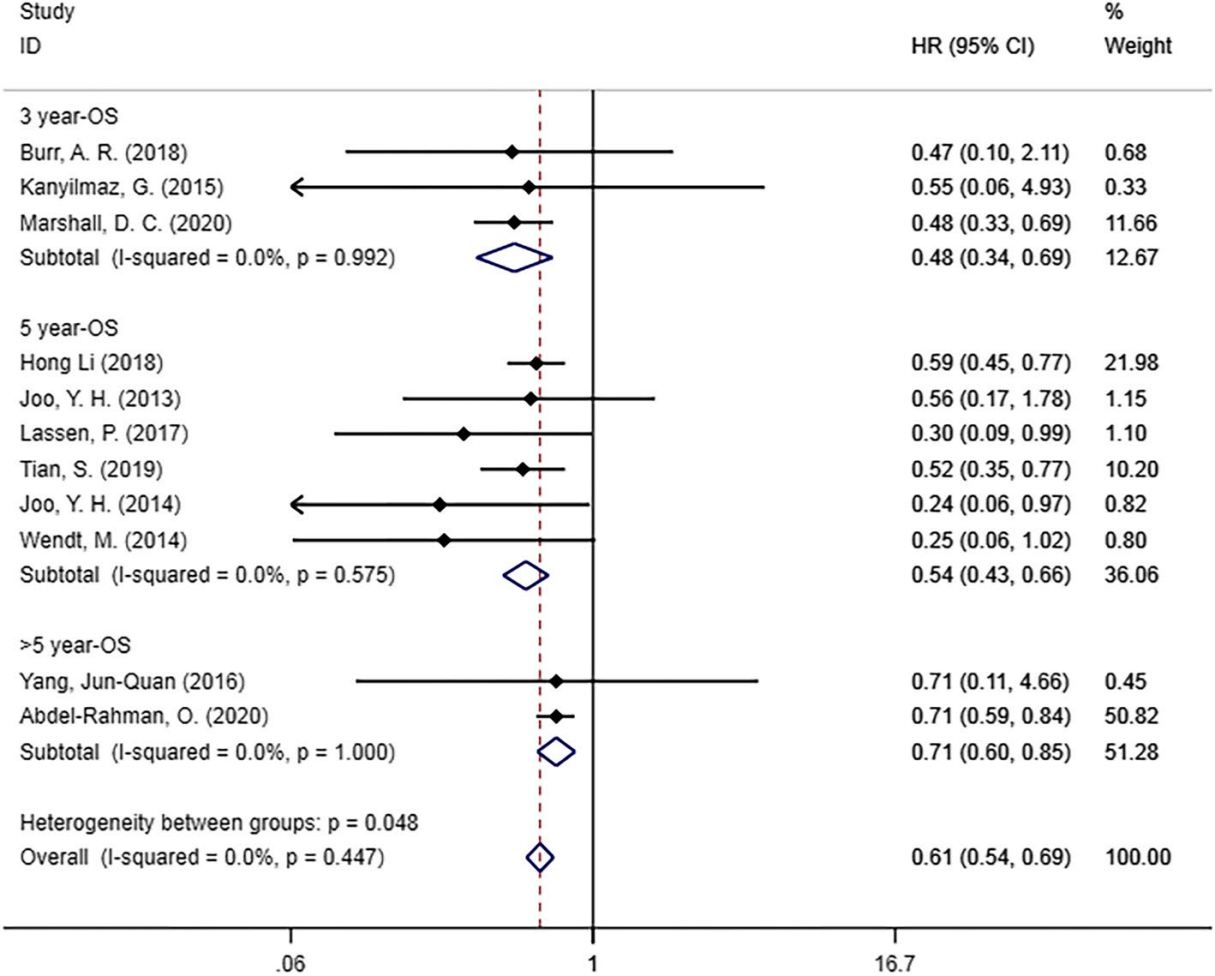
Forest plot of OS in patients with HPV DNA-positive hypopharyngeal cancer compared with HPV DNA-negative

Eleven studies [17–20, 25–31] examined the OS of patients with HPC (Fig. 2). We found that there was a statistically significant difference in OS between the HPV DNA-positive and HPV DNA-negative groups (HR = 0.61, 95% CI [0.54, 0.69], *p =* 0.0001), and HPV infection was beneficial to the survival of hypopharyngeal cancer patients. In addition, we performed subgroup analysis based on survival outcomes in different years, and statistically significant differences were found in the subgroup of 3-year OS (HR = 0.48, 95% CI [0.34–0.69], *p =* 0.0001), 5-year OS (HR, 0.54; 95% CI, 0.43–0.66; *p =* 0.0001) and > 5-year OS (HR = 0.71, 95% CI [0.60–0.85], *p =* 0.0001).

#### Disease-free survival

**Fig. 3:**
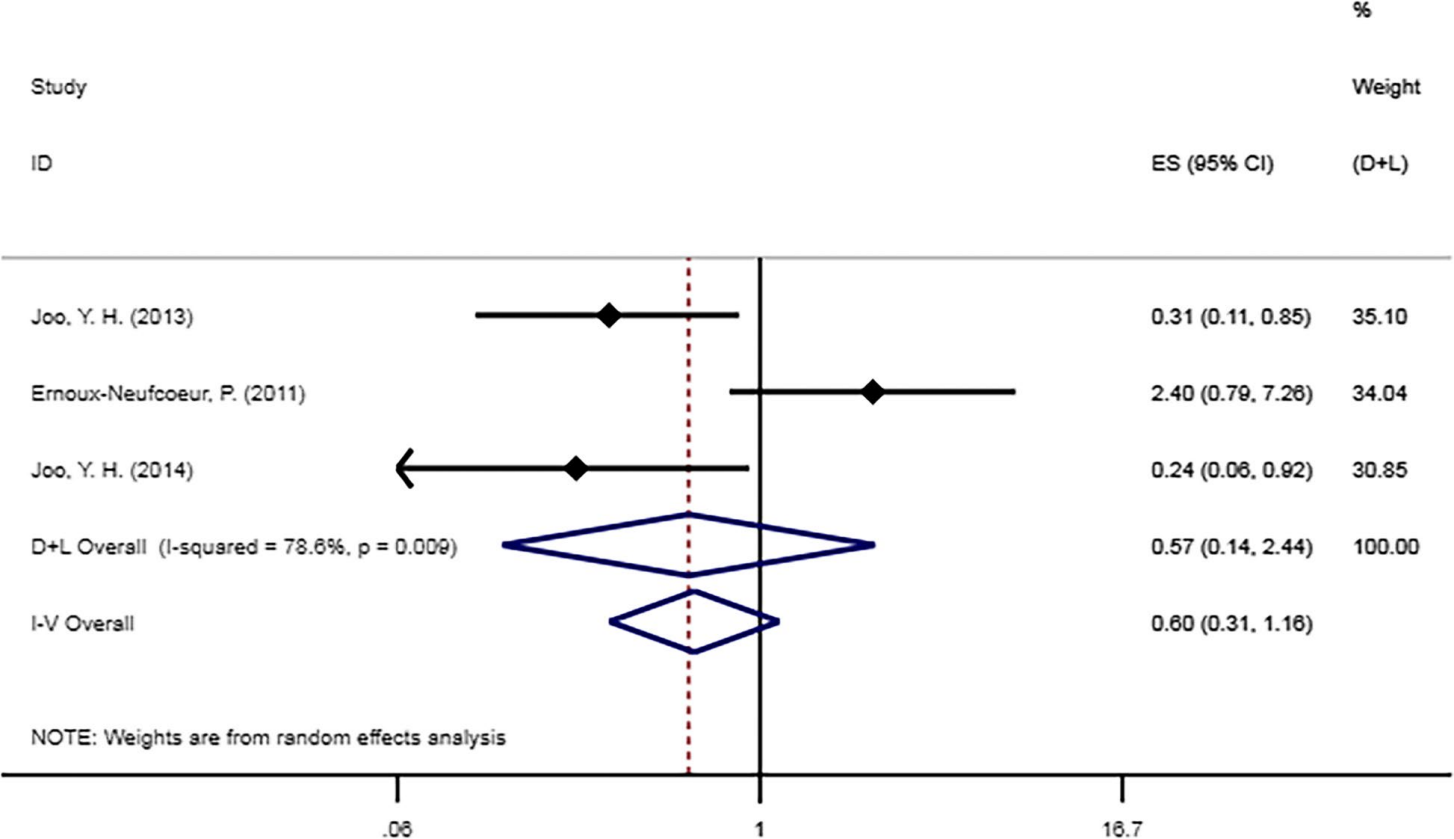
Forest plot of DFS in patients with HPV DNA-positive hypopharyngeal cancer compared with HPV DNA-negative

Three studies [18, 21, 30] examined 5-year DFS for patients with HPC (Fig. 3). There was no statistically significant difference in 5-year DFS between the HPV-negative and HPV DNA-positive groups (HR = 0.60, 95% CI [0.31–1.16], *p* = 0.13).

### Survival according to p16 status

#### Overall survival

**Fig. 4:**
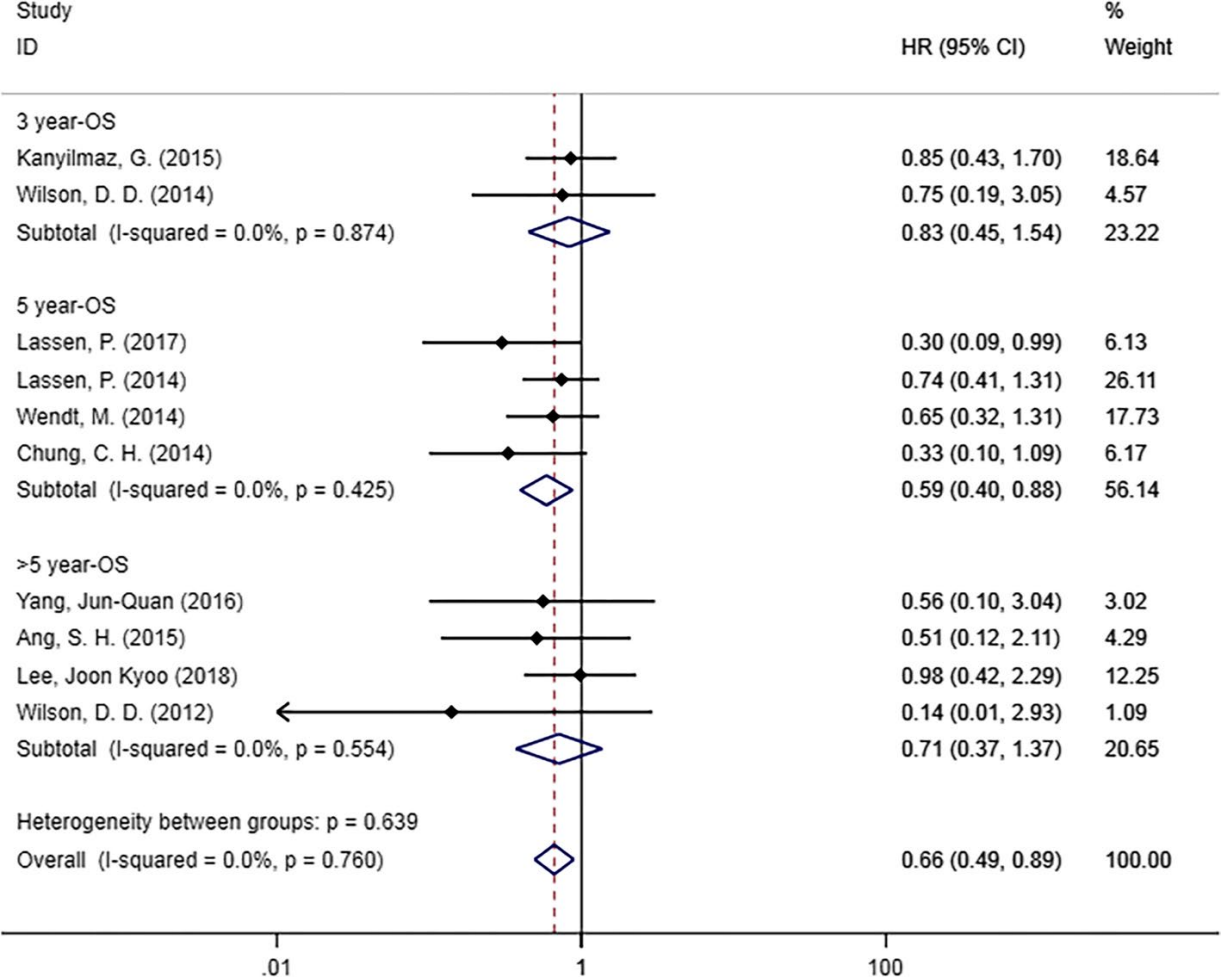
Forest plot of OS in patients with p16-positive hypopharyngeal cancer compared with p16-negative

Ten studies [20, 25, 28, 29, 32–37] examined the relationship between OS and p16 status (Fig. 4). In the meta-analysis, we found that HPC patients who were p16-positive had a significantly superior DFS compared with p16-negative patients (HR = 0.66, 95% CI [0.49–0.89], *p* = 0.007). In the subgroup analysis by survival outcomes in different years, significant differences were found for 5-year OS in the subgroup (HR = 0.59, 95% CI [0.40–0.88]; *p* = 0.009). However, no statistically significant differences were found in 3-year OS (HR = 0.83, 95% CI [0.45–1.54], *p* = 0.551) or > 5-year OS (HR = 0.71, 95% CI [0.37–1.37], *p* = 0.307).

#### Disease-free survival

**Fig. 5:**
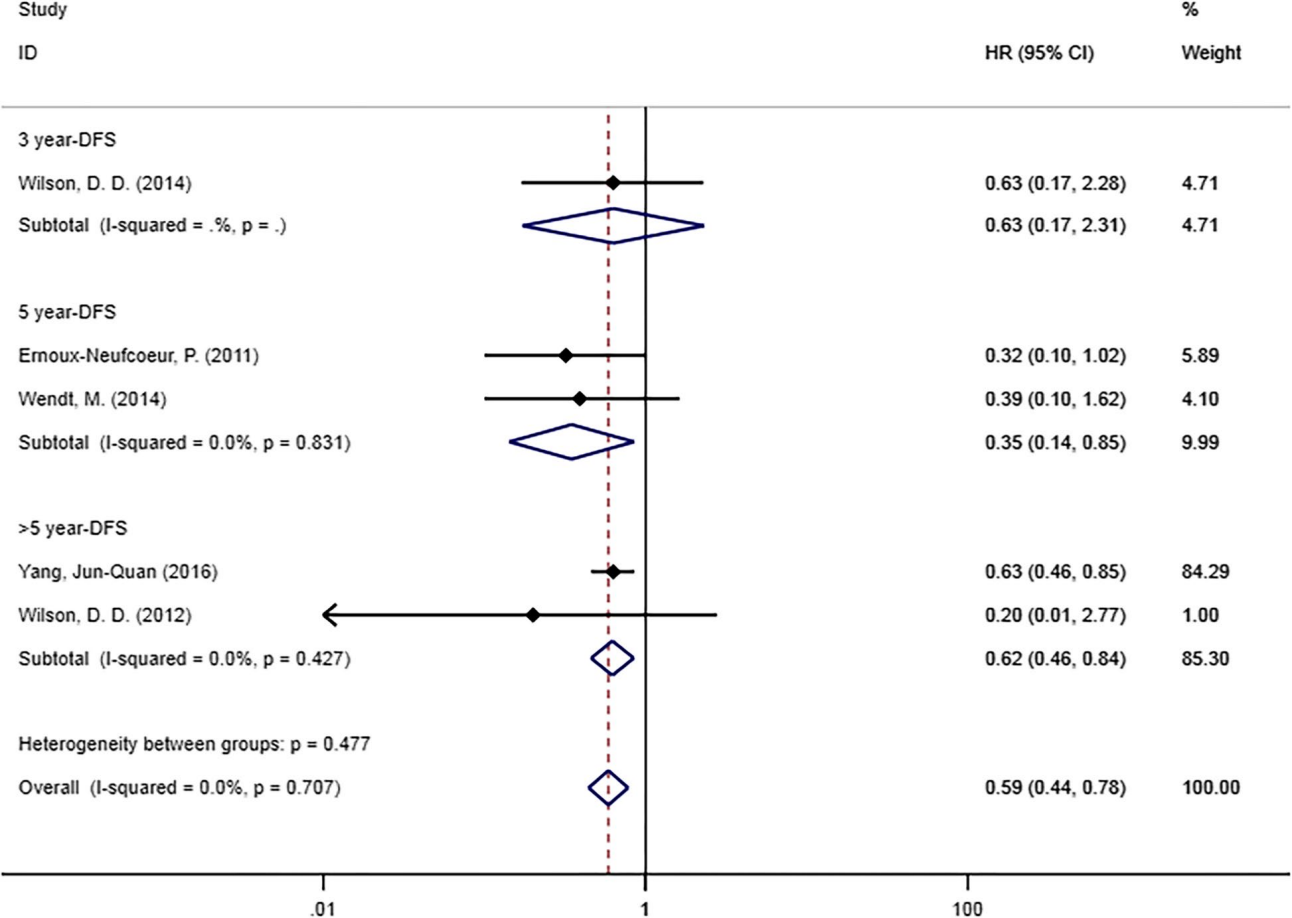
Forest plot of DFS in patients with p16-positive hypopharyngeal cancer compared with p16-negative

Five studies [20, 21, 25, 32, 33] reported DFS according to p16 status (Fig. 5). DFS was significantly associated with p16 positivity (HR = 0.59, 95% CI [0.44–0.78], *p* = 0.001), and p16 infection was beneficial to the survival of HPC patients. In the subgroup analysis by survival outcomes, significant differences were found in the subgroup of 5-year DFS (HR = 0.35, 95% CI [0.14–0.85, *p* = 0.02) and > 5-year DFS (HR = 0.62, 95% CI [0.46–0.84], *p* = 0.002). No statistically significant difference was found in the subgroup of 3-year DFS (HR = 0.63, 95% CI [0.17–2.31], *p* = 0.485). Since there was only one study in this group, the relationship between DFS and p16 status remains to be further explored.

### The publication bias of the included studies

Publication bias was evaluated by funnel plots and Egger’s test, as shown in Table 3. Publication biases were observed in the studies reporting OS according to p16 status and HPV DNA status. However, there was no significant difference in the HRs before and after trim and fill. Therefore, the risk of publication bias was considered low, and the results were stable.

**Table 3:** Publication bias (Egger test) and sensitivity analysis (trim and fill method) performed for included studies

	Egger test (*t,P*)	Number of trim and fill	HR (95%CI), *P*^*a*^	HR (95%CI), *P*^*b*^
HPV DNA-OS	-2.75,0.023	0	0.610(0.538,0.692),0.0001	0.610(0.538,0.692) ,0.0001
HPV DNA-DFS	-0.23,0.855	-	0.601(0.311,1.161),0.130	-
p16-OS	-2.47,0.039	0	0.663(0.493,0.892),0.007	0.663(0.493,0.892),0.007
p16-DFS	-2.53,0.086	-	0.587(0.443,0.778),0.0001	-

*Abbreviations: HPV DNA *Human papilloma virus DNA, *OS *overall survival (OS), *DFS *disease-free survival

^a^ Original variation. ^b^ Variation after trim and fill

## Discussion

Multiple studies have highlighted the prognostic significance of HPV status in some HNSCCs, while studies on the relationship between HPV and the prognosis of hypopharyngeal carcinoma have failed to reach a unified conclusion after years of research. Currently, in various HPC studies, HPV has been detected in a variety of ways. At the DNA level, PCR and ISH are often used to detect HPV DNA. At the protein level, p16 is used as the main detection indicator, and p16 is usually detected by IHC. In this study, both of these levels were included to comprehensively evaluate the effect of HPV infection in hypopharyngeal carcinoma.

At present, many studies have examined the relationship between HPV infection and survival indicators such as OS and DFS. OS is considered to be the best endpoint to measure tumor efficacy and is the preferred survival indicator. In our study, we found that there was a statistically significant difference in OS between the HPV DNA-positive and HPV DNA-negative groups. HPV DNA has a higher predictive value for the short-term survival of patients with HPC. Axel Sahovaler’s research [38] points out that in laryngeal and hypopharyngeal locations, patients with HPV DNA-positive tumors had an improved OS. An HPV-related study of HPC was recently published [39], and the results of this article showed that survival rates for HPV DNA-positive HPC patients improved regardless of treatment, which further confirms the reliability of our research results.

DFS was defined as the length of time after treatment during which there was no disease recurrence or death due to disease progression. At one institution in the southern United States, 13% of patients with laryngeal or HPC were found to be HPV positive. This institution found that HPV infection was associated with improvements in patients’ OS and DFS, but none of these associations was statistically significant [40]. In our study, we were unable to find any difference in DFS of HPV in patients with HPC. However, in addition to Ernoux-Neufcoeur’s study [21], two other studies showed significant differences in DFS of HPV in patients with HPC, possibly because Ernoux-Neufcoeur’s study only included resectable stage IV hypopharyngeal cancers, which has a higher survival rate. In addition, there were only three articles related to DFS, so the results of this meta-analysis need to be verified by more studies. It is worth mentioning that the HPV DNA-positive group in this group was high-risk HPV positive. High-risk HPV status was also associated with prognosis in multiple non-OPSCC populations [26]. In a cohort study of primary HNSCC [41], prognosis in OPSCC patients with salivary high-risk HPV-positive have better prognosis, especially event-free survival, than those with high-risk HPV-negative. In addition, Licitra et al. [12] found that high-risk HPV-associated OPSCC had a reduced tendency to develop second tumors compared with HPV-negative tumors. He surmised that the reduction in carcinogens exposure in patients with high-risk HPV-associated tumors reduced the occurrence of second tumors.

p16 has been a controversial prognostic factor for HPC. According to the article of Meshan et al. [42], although the survival rate of p16-positive patients was higher than that of p16-negative patients, the difference between them was not statistically significant [43]. However, many related studies have proposed that p16, as a surrogate marker of HPV, is an important prognostic marker of HNSCC and should be considered to increase the detection of p16 in the hypopharynx and other sites [44–46].

Similarly, our study found significant improvement in both OS and DFS in patients with p16-positive HPC. In the subgroup analysis of OS, only the 5-year OS was statistically significant, and the absence of statistical significance in the other two subgroups may be due to differences between studies in patient population characteristics such as age, sex distribution, race and ethnicity. The pooled HRs of all groups were less than 1, suggesting that p16 positivity had a certain beneficial effect on the OS of patients with HPC., 5-year DFS and > 5-year DFS were statistically significant, but only one of the five articles showed statistical significance. Our study suggested that p16 could improve the prognosis of DFS in patients with HPC to a certain extent, but one group of DFS subgroup analysis results was not statistically significant, so more studies are still needed to confirm the relationship between them.

Our results support the hypothesis that HPV-positive and HPV-negative hypopharyngeal carcinoma differ in relation to survival outcomes, which has also been found in other HPV-associated cancers, including laryngeal and oropharyngeal squamous cell carcinomas [12, 47]. Several studies have discussed the mechanisms by which HPV status influences the prognosis of related cancers. Studies have shown that TP53 was the most frequently mutated gene in non-HPV-associated HNSCC, and TP53 mutations were conducive to increased tumor aggressiveness [48]. However, TP53 mutations were rarely observed in HPV-positive HNSCC, and there was a specific T cell-mediated immune response in HPV-positive tumors, which significantly improves disease-specific survival [49].

Currently, there is no literature on the mechanism by which HPV infection affects the prognosis of patients with HPC, but some studies have proposed the mechanism by which HPV affects prognosis in patients with HNSCC, suggesting that HPV-positive cancers may have a lower degree of serious genetic changes than HPV-negative cancers. Because of impaired DNA repair ability and radiation-induced immune responses, HPV-positive cancers are more sensitive to radiation than HPV-negative cancers, which could affect the response to treatment [50, 51]. One study showed that patients with HPV-positive oropharyngeal cancer responded to induction chemotherapy at a higher rate than patients with HPV-negative tumors [52].

However, our study has some limitations that should be considered. In our study, the HRs for some articles were calculated by extracting the data from Kaplan–Meier curves [24]. There may be some inaccuracies in using this method to estimate HRs, thus adding some uncertainty to the calculated HRs. In addition, both adjusted and unadjusted HRs for each study were included in our analysis, and the lack of adjustment for other prognostic factors in some HRs can also introduce bias in the assessment results. Larger studies adjusting for prognostic factors are needed in subsequent studies.

## Conclusion

The presence of HPV DNA leads to better OS in patients with hypopharyngeal cancer, and the presence of p16 plays a certain role in improving OS and DFS. To the best of our knowledge, this is the largest and most comprehensive meta-analysis of HPV DNA and p16 survival outcomes in HPC, confirming the impact of HPV infection on the prognosis of hypopharyngeal cancer and providing up-to-date evidence to clinicians and researchers.

## Supplementary Information

**Additional file 1.**

## Data Availability

All data generated or analysed during this study are included in this published article (and its Supplementary Information files).
