# Supplementary material for: The effect of HPV DNA and p16 status on the prognosis of patients with hypopharyngeal carcinoma: a meta-analysis
Source: BMC Cancer. 2022 Jun 15;22:658. doi: 10.1186/s12885-022-09769-w (PMC9202146; doi:10.1186/s12885-022-09769-w)
Supplement: Supplementary file 1 — Additional file 1. [file 12885_2022_9769_MOESM1_ESM.docx]

**Additional file 1**

Table S1. HPV genotypes detected in the included studies.

| **Study** | **HPV genotypes** |
| --- | --- |
| Hong et al, 2018 | high-risk HPV types (HPV-16 and/or HPV-18) |
| Joo et al, 2013 | high-risk HPV types (HPV- 16, 18, 31, 33, 35, 39, 45, 51, 52, 56 and 66) |
| Ernoux et al, 2011 | HPV-16, 18, 33, 39, 51, 53, 58, 59 and 66 |
| Lassen et al, 2017 | HPV-16 |
| Yang et al, 2016 | HPV-16 |
| Burr et al, 2018 | high-risk HPV types (HPV-16 and/or HPV-18), low-risk HPV types |
| Dalianis et al,2015 | HPV-16 |
| Marshall et al, 2020 | NS |
| Abdel et al, 2020 | NS |
| Tian et al, 2019 | high-risk HPV types (HPV-16 and/or HPV-18), low-risk HPV types |
| Joo et al, 2014 | high-risk HPV types (HPV- 16, 18, 31, 33, 35, 39, 45, 51, 52, 56 and 66) |
| Wendt et al, 2014 | HPV-16, 51, 53 and 56 |
| Abbreviations: HPV, human papillomavirus; NS, not specified. | |

Table S2. Definition criteria for p16 positivein the included studies.

| **Study** | **Definition criteria for** **p16 positive** |
| --- | --- |
| Ernoux et al,2011 | p16 positive (strong, diffuse staining); p16 negative (weak or absent staining; i.e., the labelling index (LI) corresponding to the percentage of immunopositive cells was ≤5%). |
| Lassen et al,2017 | p16 positive: in case of strong cytoplasmatic and nuclear staining in >70% of tumor cells and in consideration of the typical microscopic appearance of an HPV-related tumor. |
| Yang et al,2016 | 10 high magnification visions were selected randomly in each stained section and 10 high power field representatives were observed, the brown nuclear staining cells were counted. Positive staining in more than 10% of the cells was considered p16 positive, while less than 10% or colorless were defined as p16 negative. |
| Dalianis et al,2015 | p16 positive: Dark brown p16 staining on at least 75% of the tumour mass. |
| Lassen et al,2014 | p16 positive: in case of strong cytoplasmatic and nuclear staining in >70% of tumor cells and in consideration of the typical microscopic appearance of an HPV-related tumor. |
| Ang et al,2015 | p16 positive: strong nuclear and cytoplasmic staining in ≥70% tumor cells |
| Lee et al,2018 | p16 positive: Staining intensity >3 ( 0, negative; 1, weakly positive; 2, moderately positive; 3, strongly positive). |
| Wilson et al,2012 | p16 positive: strong nuclear and cytoplasmic staining was present in more than 60% of tumor cells |
| Wilson et al,2014 | p16 positive: a cervical specimen with a high-grade squamous intraepithelial lesion served |
| Wendt et al,2014 | p16 positive: Dark brown p16 staining on at least 75% of the tumour mass. |
| Chung et al,2014 | p16 positive: defined as strong and diffuse nuclear and cytoplasmic staining in ≥ 70% of the tumor cells |
| Abbreviations: HPV, human papillomavirus; NS, not specified. | |
